# Drug-induced interstitial lung disease caused by olaparib: three case reports and review of the Japanese Adverse Drug Event Report database and literature

**DOI:** 10.1186/s12890-023-02569-3

**Published:** 2023-08-08

**Authors:** Hiroshi Ishimoto, Noriho Sakamoto, Takashi Kido, Mutsumi Ozasa, Shin Tsutsui, Mayako Mori, Daichi Setoguchi, Shinnosuke Takemoto, Yasushi Obase, Yuji Ishimatsu, Chiharu Tomonaga, Kanako Matsumoto, Sachiko Morisaki, Kiyonori Miura, Hiroshi Mukae

**Affiliations:** 1 Department of Respiratory Medicine, Nagasaki University Graduate School of Biomedical Sciences, 1-7-1 Sakamoto, Nagasaki, 852-8501 Japan; 2 Department of Pathology, Nagasaki University Graduate School of Biomedical Sciences, 1-7-1 Sakamoto, Nagasaki, 852-8501 Japan; 3 Department of Radiology, Nagasaki University Graduate School of Biomedical Sciences, 1-7-1 Sakamoto, Nagasaki, 852-8501 Japan; 4 Department of Nursing, Nagasaki University Graduate School of Biomedical Sciences, 1-7-1 Sakamoto, Nagasaki, 852-8520 Japan; 5 Department of Obstetrics and Gynecology, Nagasaki University Graduate School of Biomedical Sciences, 1-7-1 Sakamoto, Nagasaki, 852-8501 Japan

**Keywords:** Olaparib, Drug-induced interstitial lung disease, Hypersensitivity pneumonitis like pattern, Ovarian cancer, PARP inhibitor, Case report

## Abstract

**Background:**

Olaparib, a poly (ADP-ribose) polymerase (PARP) inhibitor, has demonstrated effectiveness in treating ovarian, breast, and other cancers, particularly those with specific molecular subtypes including, but not limited to, BRCA1/2 mutations. Consequently, its utilization is expected to increase in the future. For this reason, it is important to acknowledge the potential for adverse events associated with olaparib, including the relatively rare but significant risk of drug-induced interstitial lung disease (DIILD). Since DIILD can lead to fatal outcomes, its early detection is crucial. The dissemination of knowledge regarding DIILD can be facilitated through case reports; however, specific reports of DIILD caused by olaparib have only been published in Japanese. To the best of our knowledge, this is the first report in English of our experience with three cases of DIILD caused by olaparib.

**Case presentation:**

Cases 1, 2, and 3 involved Japanese women with ovarian cancer who had been receiving olaparib at a dose of 600 mg/day. Case 1, a 72-year-old woman who had been on olaparib for 4 months, and case 2, a 51-year-old woman who had been on olaparib for 8 months, reported fever and general malaise. Chest computed tomography (CT) revealed pale ground glass opacity (GGO) similar to hypersensitivity pneumonitis. The severity grade was 2 in both cases. Case 3, a 78-year-old woman who had been on olaparib for 3 weeks, presented with cough and reported dyspnea on exertion. Chest CT revealed non-specific interstitial pneumonia and organizing pneumonia-like shadows. The severity grade was 4. Olaparib was discontinued in all cases. Case 1 received 0.6 mg/kg of prednisolone due to mild hypoxia, while prednisolone was not administered in case 2 due to the absence of hypoxia. Case 3 received steroid pulse therapy due to severe hypoxia. Olaparib administration was not resumed in any patient.

**Conclusion:**

DIILD caused by olaparib in Japan, including the present three cases, commonly presents with GGO, similar to hypersensitivity pneumonitis on chest CT. The prognosis for the majority of patients is favorable; however, there have been instances of severe cases. Early recognition of drug-induced lung injury and further accumulation of cases is important.

## Background

Poly (ADP-ribose) polymerase (PARP) is involved in the repair of single-strand DNA breaks. Olaparib, as a PARP inhibitor, exerts anti-tumor effects by inhibiting this repair mechanism, thereby inducing cell death through synthetic lethality in tumors with pre-existing deficient double-stranded DNA repair mechanisms, such as BRCA1/2 mutations and other homologous recombination-related gene alterations. Olaparib, together with similar compounds like niraparib and rucaparib, is indicated for the treatment of various solid tumors, such as ovarian, breast, pancreatic, and prostate cancers. The selection of molecular targets of deficient DDR mechanisms varies depending on the indications and often encompasses alterations of DDR genes beyond BRCA1/2, broadly referred to as BRCAness or PARPness features [1, 2]. A systematic review using randomized controlled studies showed that PARP inhibitors increased the risk of pneumonitis (Peto odds ratio 2.68 [95% confidence interval 1.31–5.47], *p* = 0.007) [3]. Drug-induced interstitial lung disease (DIILD) attributed to anticancer drugs represents the most common type of DIILD, accounting for 23–51% of all cases [4]. Thus, the potential occurrence of DIILD must be considered during systemic treatment for malignant neoplasms. However, there have been only a limited number of reported cases of DIILD associated with PARP inhibitors. Notably, all reported cases of DIILD were linked to olaparib and were published in Japanese literature [5, 6]. To the best of our knowledge, there are no reports in English, and we have detailed the clinical course of three cases of DIILD due to olaparib.

## Case presentation

### Case 1

**Fig. 1:**
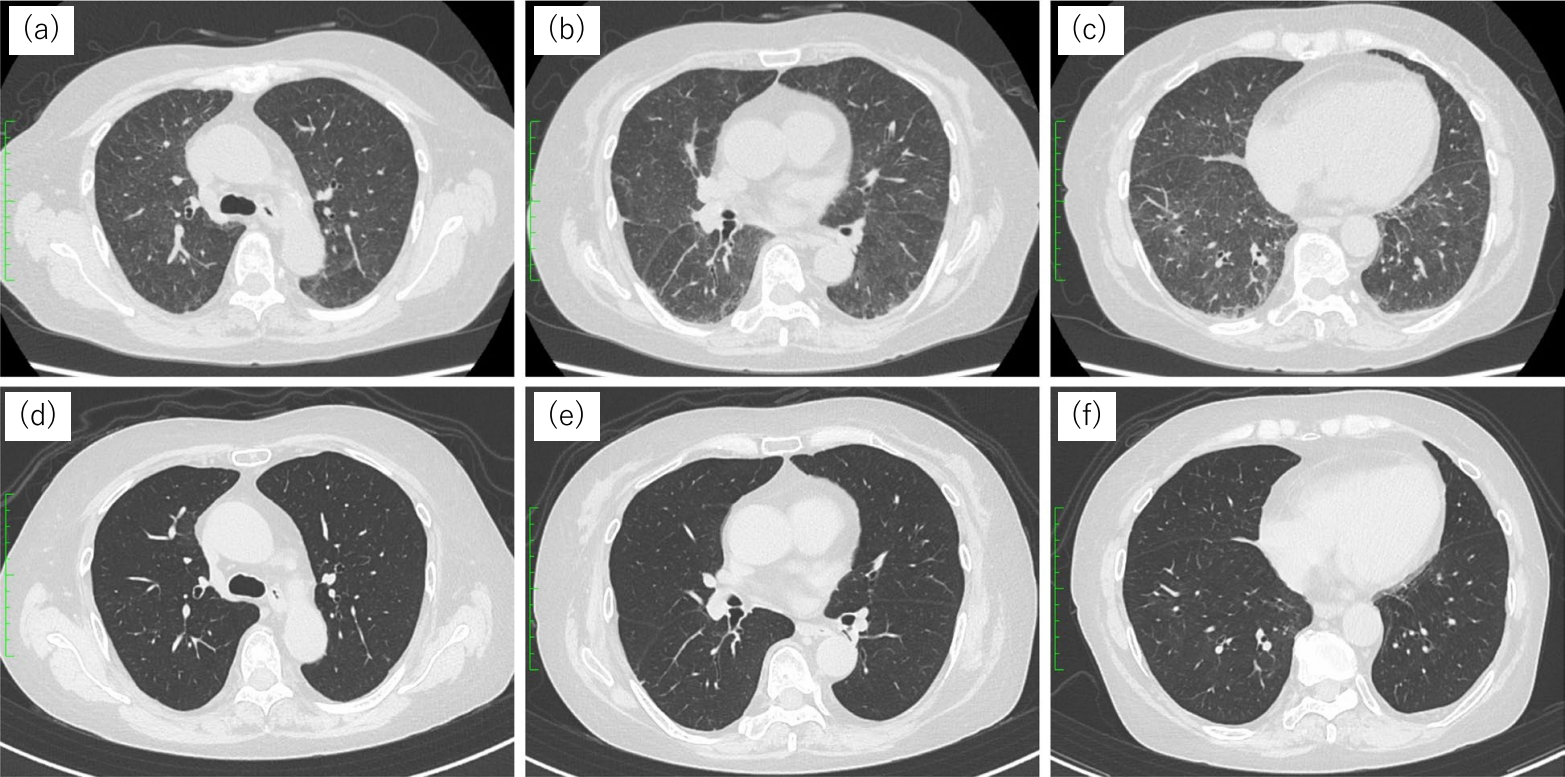
High-resolution computed tomography of the chest at the time of diagnosis in case 1, showing diffuse faint ground-glass opacity with mid- and lower-lobe predominance in both lungs (**a**-**c**). One month later, complete resolution of the shadows is observed (**d**-**f**)

A 72-year-old Japanese woman had continued prednisolone 10 mg/day for myelodysplastic syndrome for 15 years. In addition, she had been receiving zolpidem tartrate, esomeprazole magnesium hydrate, and amlodipine besylate for more than 10 years. One year prior, she was diagnosed with ovarian cancer and received a total of six courses of chemotherapy with carboplatin, paclitaxel, and bevacizumab before and after surgery. Subsequently administration of olaparib in combination with bevacizumab was initiated. Olaparib administration was continued for four months, during which her performance status (PS) was scored as 1. Olaparib was administered at 600 mg/day. Subsequently, after two weeks of fever and fatigue, she was admitted to our hospital. And chest computed tomography (CT) revealed faint ground glass opacity (GGO) resembling hypersensitivity pneumonitis in both lungs (Fig. 1a-c). Oxygen saturation in room air was 95%, but the lung sounds were not abnormal. The results of blood tests are shown in Table 1.

**Table 1:** Blood test result on admission of case 1

**Laboratory findings**		**Reference value**
WBC (×103/μL)WBC (×103/μL)	6.9	3.3 - 8.6
Seg (%)	70.8	38 - 74
Lymph (%)	13.7	16.5 - 49.5
Mono (%)	13.7	2 - 10
Eosino (%)	0.4	0 - 8.5
RBC (×106μL)RBC (×106μL)	3.27	4.35 - 5.55
Hg (g/dL)	10.6	13.7 - 16.8
Hct (%)	32.1	40.7 - 50.1
Plt (μL)	135	158 - 348
TP (g/dL)	5.6	6.6 - 8.1
Alb (g/dL)	2.8	4.1 - 5.1
AST (IU/L)	16	13 - 30
ALT (IU/L)	10	10 - 42
LDH (IU/L)	256	124 - 222
BUN (mg/dL)	11	8 - 20
Cr (mg/dL)	0.91	0.65 - 1.07
CRP (mg/dL)	4.8	0.00 - 0.14
KL-6 (U/mL)	754	105.3 - 401.2
Anti-nuclear antibodies (n times)	< 80	< 80
Anti-ARS antibody (INDEX)	< 5.0	< 25
Anti-CCP antibody (U/mL)	< 0.6	< 4.5
Anti-SS-A antibody (U/mL)	3.4	< 10
Anti-SS-B antibody (U/mL)	< 1.0	< 10
MPO-ANCA (U/mL)	< 1.0	< 3.5
PR3-ANCA (U/mL)	< 1.0	< 3.5
Beta D-Glucan (pg/mL)	20.5	< 20
Cytomegalovirus antigen C7HRP	negative	

**Fig. 2:**
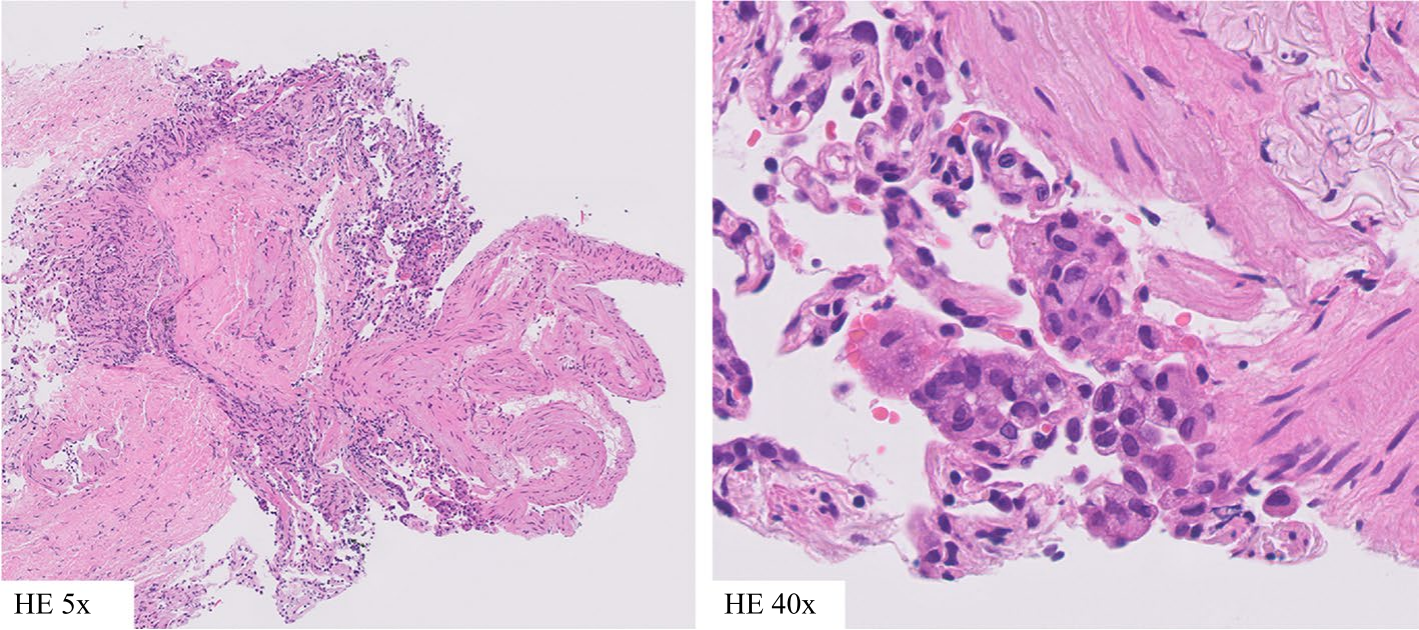
Pathological findings of transbronchial lung biopsies performed in the right upper and lower lobes in case 1. Infiltration of inflammatory cells and aggregation of foamy macrophages were observed in the alveolar space, but no specific findings were observed

There were no significant abnormalities in the blood cell counts, but C-reactive protein and Krebs von den Lungen-6 (KL-6) were elevated to 4.80 mg/dl and 754 U/ml. In addition to bronchoscopy, bronchoalveolar lavage was performed on the right middle lobe, specifically in the right B^5^ bronchus. There was an increase in the total cell count to 7.75 × 10^5^/mL, alveolar macrophages, lymphocytes, and neutrophils were 13%, 81%, and 6%, respectively, and the CD4/CD8 ratio was 3.1. No bacterial colonies were detected in the alveolar lavage fluid culture. Transbronchial lung biopsy revealed no specific findings, although there was an infiltration of inflammatory cells and aggregation of foamy macrophages in the alveolar space (Fig. 2). There were no findings suggestive of infection, and a diagnosis of DIILD was made. Olaparib and bevacizumab were discontinued. The disease severity was determined to be grade 2 based on the presence of accompanying symptoms. Therefore, olaparib was suspended and prednisolone was started at 35 mg as an equivalent of 0.6 mg per kg of body weight. As a result, the fever subsided and an improvement in their general condition was observed. Subsequently, prednisolone was gradually tapered off over a period of 3 months until it was completely discontinued. Follow-up imaging demonstrated resolution of the lung lesions (Fig. 1d-f), and no recurrence was observed.

### Case 2

A 51-year-old Japanese woman was started on olaparib 600 mg/day as a fourth-line treatment for postoperative recurrence of ovarian cancer. Her PS was scored as 1. Nine months after starting olaparib, she developed general malaise and persistent fever (38 °C). Her oxygen saturation was 99% in room air, with no hypoxia, and her lung sounds were normal. Blood tests revealed bicytopenia and elevated interstitial pneumonia marker levels (Table 2).

**Table 2:** Blood test result at the time of consultation of case 2

**Laboratory findings**		**Reference value**
WBC (×103/μL)WBC (×103/μL)	3	3.3 - 8.6
Seg (%)	81	38 - 74
Lymph (%)	7.9	16.5 - 49.5
Mono (%)	6.3	2 - 10
Eosino (%)	4.7	0 - 8.5
RBC (×106μL)RBC (×106μL)	3.01	4.35 - 5.55
Hg (g/dL)	10.1	13.7 - 16.8
Hct (%)	30.4	40.7 - 50.1
Plt (μL)	243	158 - 348
AST (IU/L)	18	13 - 30
ALT (IU/L)	16	10 - 42
LDH (IU/L)	261	124 - 222
BUN (mg/dL)	13	8 - 20
Cr (mg/dL)	0.64	0.65 - 1.07
CRP (mg/dL)	4.42	0.00 - 0.14
KL-6 (U/mL)	715	105.3 - 401.2
SP-D (ng/mL)	182	< 110
Anti-nuclear antibodies (n times)	< 80	< 80
Anti-ARS antibody (INDEX)	< 5.0	< 25
MPO-ANCA (U/mL)	< 1.0	< 3.5
PR3-ANCA (U/mL)	< 1.0	< 3.5
Beta D-Glucan (pg/mL)	17.1	< 20
Cytomegalovirus antigen C7HRP	negative	

**Fig. 3:**
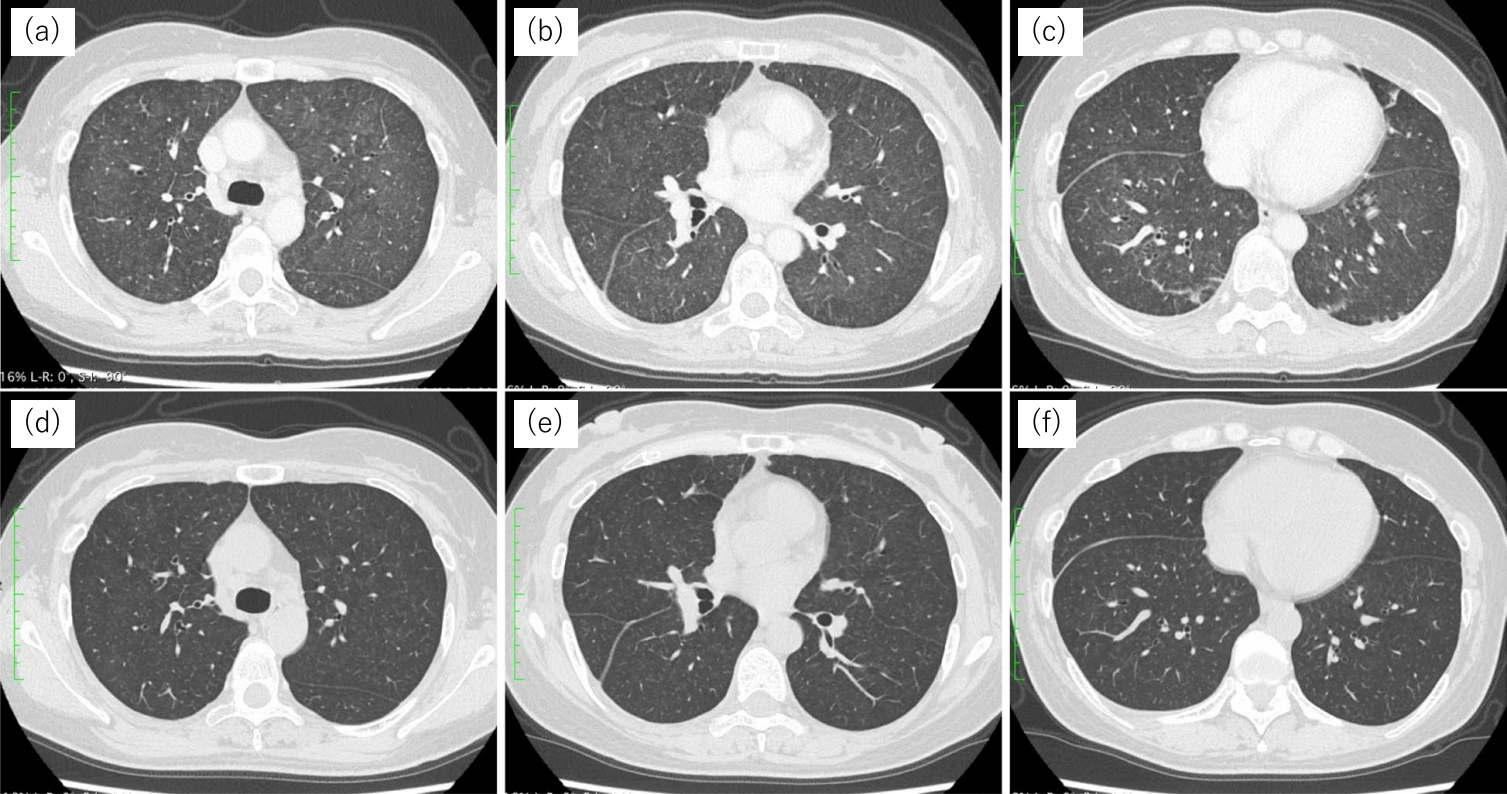
High-resolution computed tomography images of the chest at the time of diagnosis in case 2. The images show bilateral, diffuse ground-glass opacity with the presence of centrilobular ground-glass nodules. One month later, complete resolution of the shadows is observed (**d**–**f**)

In addition to olaparib, she had been receiving flunitrazepam for over a year. Chest CT revealed the presence of diffuse GGO in both lungs, similar to the pattern observed in hypersensitivity pneumonitis (Fig. 3a–c). Although bronchoscopy was not performed, DIILD was also suspected. The patient's general condition was good, and after discontinuation of olaparib without steroids or antibacterial agents, the fever subsided, and an improvement in fatigue was observed. In addition, the GGO in the lung fields improved (Fig. 3d–f), and there were no recurrences. Based on the improvement of symptoms and imaging findings following the discontinuation of olaparib, the patient was diagnosed with DIILD attributed to olaparib. The disease severity was determined to be grade 2.

### Case 3

A 78-year-old Japanese woman started olaparib at 600 mg/day as the 6th line treatment for postoperative recurrence of ovarian cancer that had been operated on six years earlier. PS was scored as 2. She had been receiving amlodipine besilate for five years. Two weeks after starting olaparib, the patient developed cough and dyspnea on exertion. Three weeks after starting olaparib, the patient was hospitalized because of fever and dyspnea. The patient had a body temperature of 38.3 °C, respiratory rate of 28 breaths per minute, and severe hypoxemia with an oxygen saturation of 88%, even after nasal cannula inhalation of 4 L of oxygen per minute. Lung sounds were heard as coarse crackles on the left lung. Blood tests revealed marked myelosuppression and high levels of inflammation (Table 3).

**Table 3:** Blood test result on admission of case 3

**Laboratory findings**		**Reference value**
WBC (×103/μL)WBC (×103/μL)	2.9	3.3 - 8.6
Seg (%)	81	38 - 74
Lymph (%)	16	16.5 - 49.5
Mono (%)	2.4	2 - 10
Eosino (%)	0.3	0 - 8.5
RBC (×106μL)RBC (×106μL)	1.78	4.35 - 5.55
Hg (g/dL)	5.3	13.7 - 16.8
Hct (%)	15.4	40.7 - 50.1
Plt (μL)	70	158 - 348
TP (g/dL)	5.8	6.6 - 8.1
Alb (g/dL)	3	4.1 - 5.1
AST (IU/L)	22	13 - 30
ALT (IU/L)	8	10 - 42
LDH (IU/L)	309	124 - 222
BUN (mg/dL)	24	8 - 20
Cr (mg/dL)	1.02	0.65 - 1.07
CRP (mg/dL)	10.82	0.00 - 0.14
KL-6 (U/mL)	356	105.3 - 401.2
SP-A (ng/mL)	140.2	< 43.8
SP-D (ng/mL)	231	< 110
Anti-nuclear antibodies (n times)	< 80	< 80
Anti-ARS antibody (INDEX)	< 5.0	< 25
Anti-CCP antibody (U/mL)	< 0.6	< 4.5
Anti-SS-A antibody (U/mL)	1	< 10
Anti-SS-B antibody (U/mL)	< 1.0	< 10
MPO-ANCA (U/mL)	< 1.0	< 3.5
PR3-ANCA (U/mL)	< 1.0	< 3.5
Procalcitonin (ng/mL)	0.136	< 0.05
Beta D-Glucan (pg/mL)	5.5	< 20
Cytomegalovirus antigen C7HRP	negative	

**Fig. 4:**
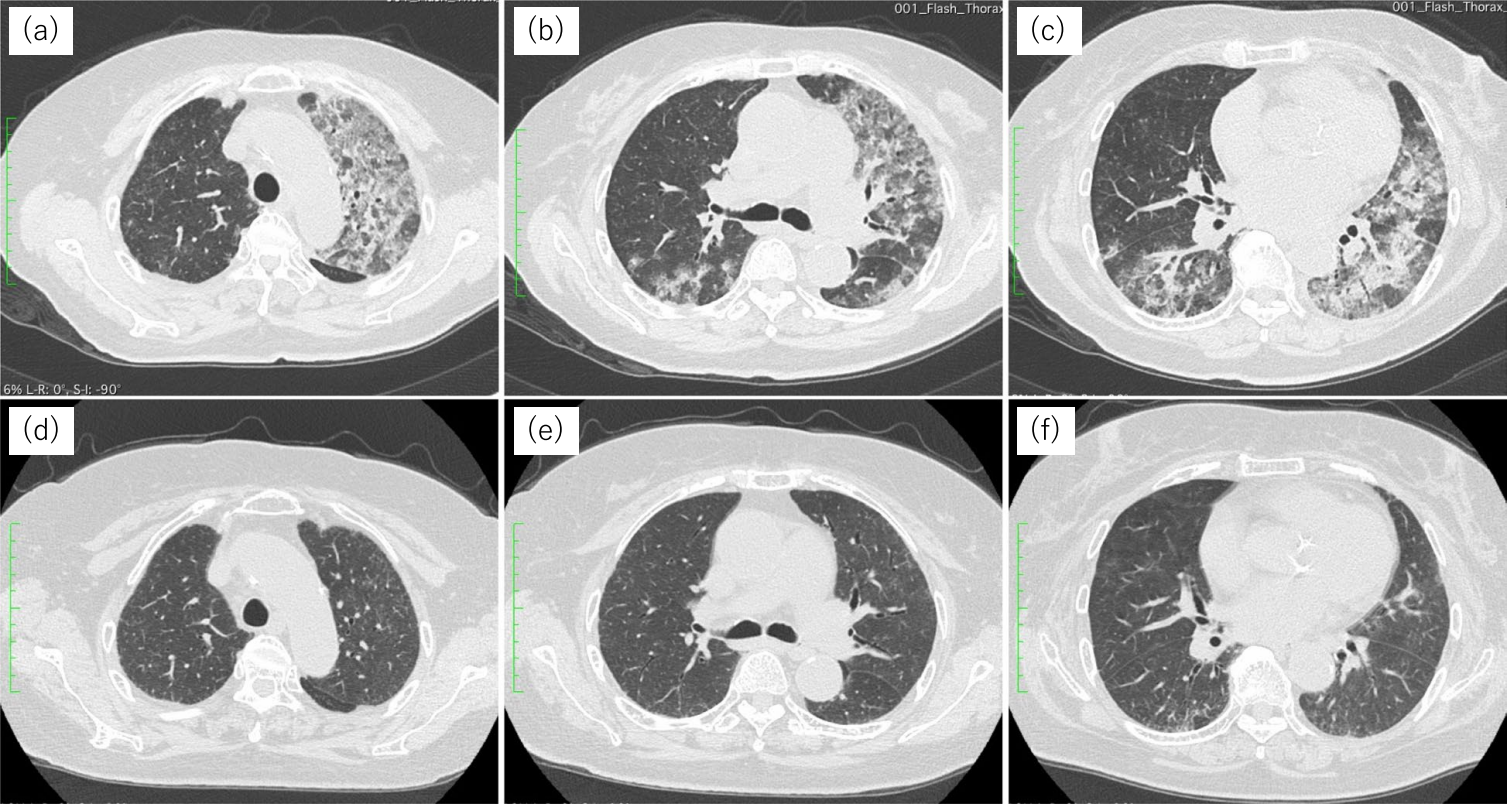
High-resolution computed tomography images of the chest at the time of diagnosis in case 3. The images show frosted shadows and consolidations in the left lung and right lower lobe. The right upper lobe, which exhibits fewer abnormal shadows, shows faint nodular shadows with a central lobular distribution and ground-glass shadows as well. Two months later, a reduction in the shadows is observed (**d**–**f**)

High-flow nasal cannula therapy was initiated, and the arterial blood oxygen partial pressure was 74.7 torr at 50% inspired air oxygenation, resulting in a PaO2/FiO2 ratio of 149.4. Antigen testing for influenza was negative. Since the patient had sought treatment prior to the outbreak of the COVID-19 pandemic, testing for COVID-19 was not performed. Urinary antigen tests for *Streptococcus pneumoniae* and *Legionella* were negative as well. Chest radiography revealed the presence of consolidation with air bronchograms in the left lung. Chest CT revealed the presence of patchy GGO and consolidation, resembling the patterns observed in nonspecific interstitial pneumonia and organizing pneumonia, affecting the left upper lobe and bilateral lower lobes (Fig. 4a–c). Bronchoscopy was not performed because of the severity of the respiratory failure. Based on the patient's clinical course and laboratory findings, the diagnosis of DIILD was considered, olaparib was discontinued, and steroid pulse therapy with methylprednisolone at 1000 mg/body was initiated. Although there were no laboratory findings to suggest opportunistic infections, the patient also had a decreased white blood cell count, and it was decided to administer antimicrobial chemotherapy with tazobactam/piperacillin and azithromycin. After a 3-day steroid pulse therapy, treatment was continued with prednisolone 60 mg/day, which resulted in the resolution of fever, improvement of oxygenation, and gradual improvement of lung field shadows. Sputum culture did reveal the presence of any bacteria, and no test results were indicative of infection. Echocardiography did not reveal any abnormalities in cardiac function. Thus, based on the successful response to steroid therapy, the patient was diagnosed with DIILD. The disease severity was determined to be grade 4. Prednisolone was tapered off, and prednisolone was terminated after approximately six months, but there were no relapses of lung shadows (Fig. 4d–f).

## Discussion and conclusion

Here, we report three Japanese cases of olaparib-induced lung disease. This is the first English report describing the details of the clinical course of DIILD caused by olaparib in Japanese patients and reviewing the Japanese Adverse Drug Event Report database and literature.

The incidence of pneumonitis, including all severities, caused by PARP inhibitors is reported to be 0.79%, consisting of olaparib, niraparib, and veliparib at 0.97%, 0.59%, and 1.00%, respectively [3]. The FDA Adverse Event Reporting System (FAERS) database reported 13 deaths (16%) of 79 cases in patients with PARP inhibitor-induced pneumonitis [3]. In using PARP inhibitors, due to the high mortality rate of DIILD caused by PARP inhibitors, it is imperative to pay adequate attention to DIILD.

DIILD is reported more frequently in Asia, including in the Japanese population [7]. We investigated cases of DIILD attributed to olaparib over four years, from 2018 (the year olaparib was approved for use by the Japanese Ministry of Health, Labour and Welfare) through 2021, on the official website of the Japanese Pharmaceuticals and Medical Devices Agency (PMDA) [http://www.info.pmda.go.jp/fsearchnew/jsp/menu_fukusayou_base.jsp] (Table 4).

**Table 4:** 110 cases of drug-induced lung disorder due to olaparib from PMDA database from 2018 to 2021

Sex	Male	2	(1.8)	Event	Interstitial lung disease	98	(89.1)
[ n (%)]	Female	108	(98.2)	[ n (%)]	Lung failure	5	(4.5)
					Pneumonitis	3	(2.7)
Age	30s	1	(0.9)		Pulmonary toxicity	3	(2.7)
[ n (%)]	40s	8	(7.3)		Alveolar hemorrhage	1	(0.9)
	50s	19	(17.3)				
	60s	27	(24.5)	Time to event	median	120	
	70s	33	(30.0)	(days, n=79)	minimun	7	
	80s	6	(5.5)		maximun	740	
	unknown	16	(14.5)				
				Olaparib	Abort	94	(85.5)
Disease	Ovary	90	(81.8)	administration	Resume after abort	7	(6.4)
[ n (%)]	Peritoneum	9	(8.2)	[ n (%)]	Not changed	5	(4.5)
	Breast	7	(6.4)		unknown	4	(3.6)
	Pancreas	1	(0.9)				
	Prostate	1	(0.9)	Outcome	Recovery	39	(35.5)
	unknown	2	(1.8)	[ n (%)]	Remission	34	(30.9)
					Not recovered	2	(1.8)
					Death	2	(1.8)
					unknown	33	(30.0)

Details regarding the medications administered for DIILD could not be obtained from this database. Furthermore, the outcomes of 33 cases (30%) were unknown, which limits the ability to determine the prognosis of DIILD caused by olaparib using this data. Recovery and remission were reported for 73 cases (66%) in total; however, two cases (1.8%) each of non-recovery and death were reported as well, which cannot be disregarded. In the majority of cases olaparib was discontinued. However, there were instances where olaparib administration could have been resumed after discontinuation or continued without interruption. The subsequent clinical course of patients after the administration of olaparib was resumed, especially concerning relapse, could not be determined due to the lack of available data in the database.

Table 5 shows the clinical features of the six Japanese case reports of olaparib-induced lung disease that are currently available in the literature, including the present three cases. Two cases reported by Sakai et al., one with non-cardiogenic pulmonary edema and the other with a hypersensitivity pneumonitis-like pattern, were treated with discontinuation of olaparib alone, without the need for steroids, and were able to restart olaparib afterward [5]. Another case reported by Suzuki et al., showed GGO, which quickly improved with steroids [6] (Table 5).

**Table 5:** Clinical features of six Japanese patients with drug-induced lung disease due to olaparib

Case	Year of publication	Author	Age/Sex	Primary disease	Olaparib dose (mg/day)	Olaparib oral durations	Symptoms	Oxygenation	CT findings	Treatment method	Effect of treatment	Re-administer olaparib
1	2020	Sakai et al.	61/F	peritoneal cancer	600	4 months	fever, hemosputum, fatigue	PaO2 67.2 Torr (room air)	non-cardiogenic pulmonary edema pattern	withdrawal of Olaparib	improvement	re-administered without relapse
2	2020	Sakai et al.	53/F	ovarian cancer	600	2 months	fever	SpO2 98%(room air)	HP-like pattern	withdrawal of Olaparib	improvement	re-administered without relapse
3	2020	Suzuki et al.	34/F	breast cancer	600	7 weeks	fever, cough, sputum	SpO2 97%(room air)	faint ground glass shadow	withdrawal of Olaparib and PSL 1mg/kg	improvement	not done
4	2022	Presentcase 1	72/F	ovarian cancer	600	4 months	fever, fatigue	PaO2 73.7 Torr (room air)	HP-like pattern	withdrawal of Olaparib and PSL 0.6 mg/kg	improvement	not done
5	2022	Presentcase 2	51/F	ovarian cancer	600	8 months	fever, fatigue	SpO2 99%(room air)	HP-like pattern	withdrawal of Olaparib	improvement	not done
6	2022	Presentcase 3	78/F	ovarian cancer	600	3 weeks	cough, dyspnea on effort	PaO2 74.7 Torr (FiO2 0.5)	NSIP and OP pattern	withdrawal of Olaparib and mPSL pulse therapy	improvement	not done

*F* female, *HP* hypersensitivity pneumonitis, *NSIP* nonspecific interstitial pneumonia, *OP* organizing pneumonia

KL-6, a biomarker that is elevated in 53% of DIILD cases and correlates with diffuse alveolar damage patterns associated with poor prognosis, is considered useful for DIILD diagnosis [7]. However, the establishment of a biomarker with high specificity for the diagnosis of DIILD remains a challenge for the future [4, 8]. The following diagnostic criteria should be used when the onset of DIILD is suspected: (1) a history of administrations of a drug known to induce lung injury, (2) exclusion of other causes for the clinical manifestations [9]. The exclusion of differential diagnoses is an essential step in diagnosing DIILD [4, 9–11]. Notably, even in the presented three cases, the diagnosis of DIILD was based on the exclusion of differential diagnoses, highlighting the challenges in diagnosing DIILD in actual clinical practice. Therefore, it is important to consider the discontinuation of the suspected drug at an early stage if DIILD cannot be ruled out. PARP inhibitors are widely used to treat various cancers. It is essential to accumulate more cases of DIILD caused by PARP inhibitors, including olaparib, and to characterize the pathogenesis to develop countermeasures.

## Data Availability

Not applicable.

## Abbreviations

DIILD: Drug induced interstitial lung disease
PARP: Poly (ADP-ribose) polymerase
DDR: DNA damage repair
CT: Computed tomography
GGO: Ground glass opacity
KL-6: Krebs von den Lungen-6
PS: Performance status
